# Identification of a Signature for Predicting Prognosis and Immunotherapy Response in Patients with Glioma

**DOI:** 10.1155/2022/8615949

**Published:** 2022-08-29

**Authors:** Wei-Feng Zong, Cui Liu, Yi Zhang, Suo-Jun Zhang, Wen-Sheng Qu, Xiang Luo

**Affiliations:** ^1^Department of Neurology, Tongji Hospital, Tongji Medical College, Huazhong University of Science and Technology, Wuhan 430030, China; ^2^Neurorehabilitation Center, Beijing Rehabilitation Hospital of Capital Medical University, Beijing 100144, China

## Abstract

Glioma is a deadly tumor that accounts for the vast majority of brain tumors. Thus, it is important to elucidate the molecular pathogenesis and potential diagnostic and prognostic biomarkers of glioma. In the present study, gene expression profiles of GSE2223 were obtained from the Gene Expression Omnibus (GEO) database. Core modules and hub genes related to glioma were identified using weighted gene coexpression network analysis (WGCNA) and protein-protein interaction (PPI) network analysis of differentially expressed genes (DEGs). After a series of database screening tests, we identified 11 modules during glioma progression, followed by six hub genes (RAB3A, TYROBP, SYP, CAMK2A, VSIG4, and GABRA1) that can predict the prognosis of glioma and were validated in glioma tissues by qRT-PCR. The CIBERSORT algorithm was used to analyze the difference of immune cell infiltration between the glioma and control groups. Finally, Identification VSIG4 for immunotherapy response in patients with glioma demonstrating utility for immunotherapy research.

## 1. Introduction

Glioma is a deadly tumor that accounts for about a third of all brain tumors [1]. They originate from the glial cells in the central nervous system and have a more malignant histological appearance compared to other brain tumors [2]. The World Health Organization classifies glioma into low and high grades. Low-grade glioma (LGG) (astrocytoma, oligodendroglioma, and oligoastrocytoma) is a well-differentiated tumor with high 5-year survival rates of almost 60% [3–5]. Among the high-grade gliomas, glioblastoma (GBM) is the most aggressive tumor with a median survival of only 15 months [6]. About 70% of LGG patients progress to a high grade within 5–10 years [7].

Glioma usually occurs relatively late in life, which makes complete resection difficult [8]. Current treatment for glioma includes surgical removal, followed by chemotherapy and radiation. Aggressive surgical treatment is associated with serious side effects, including the development of resistance against chemotherapy and radiotherapy, leading to treatment failure, tumor recurrence, and ultimately death [9]. Therefore, it is necessary to identify new diagnostic and prognostic biomarkers for personalized gene and molecular therapies.

Weighted gene coexpression network analysis (WGCNA) is a systems biology method used to identify the correlations between genes in microarray samples, as well as identify modules of highly correlated genes [10]. WGCNA can be used to discover genes and biological processes of unknown function, candidate diseases, or transcriptional regulatory work. Although WGCNA cannot prove a causal relationship, coexpression networks can identify regulatory genes of different phenotypes. The network approach bridges the gap between individual genes and systemic tumors [11, 12].

In this study, the GSE2223 dataset, including 50 glioma samples and four control samples, were used to conduct WGCNA. Gene coexpression networks and gene modules were identified. Overlapping genes were evaluated using the black-blue module and protein-protein interaction (PPI) network of differentially expressed genes (DEGs) using the cytoHubba plugin to identify diagnostic and prognostic related hub genes of glioma. Finally, identification of VSIG4 for immunotherapy response in patients with glioma indicates that the VSIG4 signature may have the ability to predict the effect of immunotherapy in glioma.

## 2. Materials and Methods

### 2.1. Data Processing

**Figure 1:**
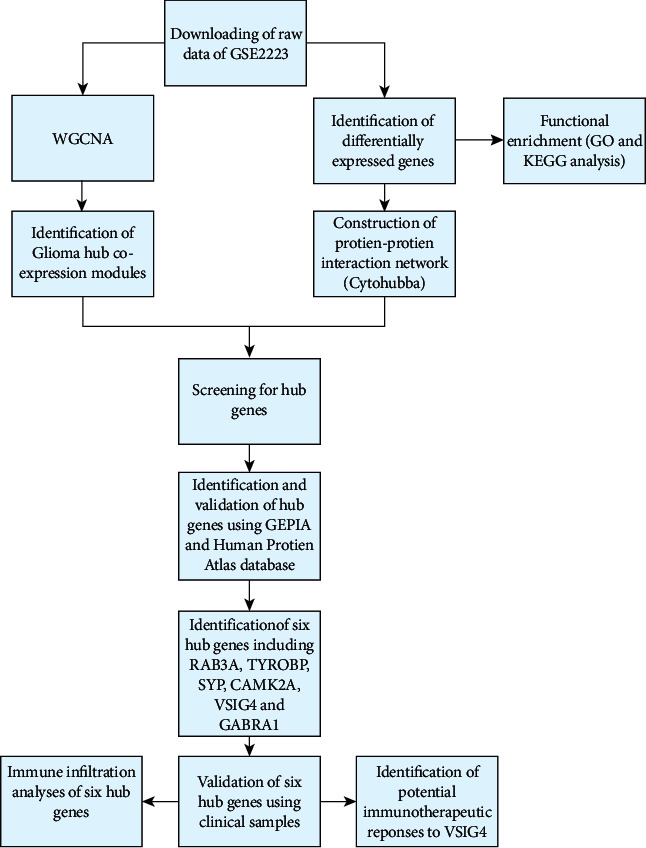
Schematic flow diagram of the study.

Figure 1 depicts the procedures used for data preparation, processing, analysis, and validation. Gene expression profiles of GSE2223 were downloaded from the GEO database (https://www.ncbi.nlm.nih.gov/geo/query/acc.cgi). The GPL1833 (HG-U133A) Affymetrix Human Genome U133A array was used to extract the expression profile of the GSE2223 dataset. The dataset consisted of 50 glioma and four control samples. The original expression profile was downloaded, followed by background correction and quantile normalization using a robust multiarray averaging (RMA) algorithm. Subsequently, mRNA expression matrices were developed for patients with glioma.

### 2.2. Screening and Analysis of DEGs

Cuffdiff was used to screen for DEGs between glioma and control samples. The threshold for DEGs was set as follows: adjusted *p* value <0.02; log2 (fold change) > 1.5 or log2 (fold change) < −1.5. The ggplot2 package in R software (R Foundation for Statistical Computing, Vienna, Austria) was used to display heat and volcano maps.

DEGs with unique biological significance were identified using Gene Ontology (GO) analysis. The Kyoto Encyclopedia of Genes and Genomes (KEGG) database was used to search for important pathways. The ClusterProfiler package in R was used for GO annotation and KEGG pathway analysis.

### 2.3. Coexpression Network Constructed Using WGCNA

The WGCNA R package was used to construct the coexpression network of all genes in glioma and control samples. The algorithm screened the top 25% of genes for further analysis. The WGCNA analysis was performed on 50 glioma and four control samples. The samples were used to calculate the Pearson correlation matrix. Weighted adjacency matrix was established using the formula amn= |cmn|*β* (where amn= adjacency between genes m and n; cmn= Pearson's correlation; *β*= soft-power threshold). Furthermore, the weighted adjacency matrix was transformed into a topological overlap metric matrix (TOM) to estimate its connectivity in the network. The average linkage hierarchical clustering method was used to construct a clustering dendrogram of the TOM matrix. The minimum gene module size was set at 30 to obtain suitable modules, and the threshold for merging similar modules was set at 0.25.

### 2.4. Hub Gene Identification Using WGCNA and PPI

The WGCNA modules that were most significantly correlated with the clinical phenotypes were identified. Then, the most relevant MEyellow and MEbrown modules were selected for subsequent analysis. To construct a PPI network, 311 DEGs were uploaded to the STRING database (https://string-db.org/) [13]. The confidence score was set at 0.9. The core modules of the PPI network were analyzed using Cytoscape software and plugin (cytoHubba) [14], and the degree scores of the first 100 genes were screened. Gene overlapping between cytoHubba and the yellow-brown modules were identified as hub genes.

### 2.5. Validation of Hub Genes

To validate the abnormal expression level of hub genes, the online database GEPIA [15] was used to analyze the hub gene expression profiles of glioma tissue and normal brain samples from the Cancer Genome Atlas (TCGA) database.

Results of the immunohistochemical staining were collected from the Human Protein Atlas to verify the protein levels of hub genes in glioma tissue and normal tissue (HPA, https://www.proteinatlas.org/). HPA is a Sweden-based program initiated in 2003, which maps human proteins in cells, tissues, and organs. We used the GeneMANIA online platform to analyze the hub genes and their networks of coexpressed genes (https://genemania.org/) [16].

### 2.6. Survival Analysis

The Kaplan–Meier curve of GEPIA [15] was used to analyze the overall survival (OS) and disease-free survival (DFS) for hub genes in TCGA glioma patients. The methods used were in accordance with the publisher's instructions. Only genes with *p* values <0.05 were considered potential prognostic genes.

### 2.7. Reverse-Transcription and Quantitative Real-Time PCR (qRT-PCR)

Glioma (*n*= 12) and normal brain (from traumatic decompression patients, *n*= 6) tissues were collected from the Neurosurgical Department of Tongji Hospital after obtaining written consent and approval from the Research Ethics Committee of Tongji Hospital (no. TJ-IBR20181111). Tissue total RNA was isolated using the Trizol reagent (Takara Bio Inc., Shiga Japan) according to the manufacturer's instructions. The RT Premix kit (Takara Bio Inc.) was used for reverse transcription. The reaction was conducted at 37°C for 15 min and at 85°C for 5 s. Quantitative real-time PCR (qRT-PCR) was performed using the real-time PCR system (Bio-Rad Laboratories, Hercules, CA, USA) and SYBR Green PCR Master Mix (Toyobo Co., Ltd., Osaka, Japan).  Table 1 lists the primer sequences.

### 2.8. Analysis of Immune Cell Infiltration

The CIBERSORT algorithm was used to evaluate the percentage of 22 immune cell types in each sample. The fraction of 22 immune cells was compared between the glioma and control groups, and the violin plot was drawn by the “vioplot” R package. The correlation coefficient between immune cells was calculated using the “corrplot” R package. Spearman correlation analysis was also performed to investigate the correlation of RAB3A, TYROBP, SYP, CAMK2A, VSIG4, GABRA1, and infiltrating immune cells.

### 2.9. Statistical Analysis

Statistical analyses were performed using GraphPad Prism (GraphPad Software, Inc., San Diego, CA, USA). Hub gene PCR data are expressed as means ± standard deviations (SDs). Nonparametric tests (Mann–Whitney *U* test) were used to compare variables between the groups. Receiver-operating characteristic curves were generated to determine the diagnostic value of hub genes. *p* values <0.05 were considered significant.

## 3. Results

### 3.1. Data Collection and DEG Analysis

Fifty glioma samples and four control samples were collected using the Glioma tissue gene chip (GSE2223). The R package “limma” was used to screen DEGs between glioma and normal samples in GSE2223; 311 DEGs were screened, including 187 downregulated genes and 124 upregulated genes. The DEGs are displayed in heat and volcano maps (Supplementary Figures 1A and 1B).

To explore the relationship between DEGs, they were annotated using GO and KEGG. The GO analysis showed that the most important GO terms were “modulation of chemical synaptic transmission” (body: biological process), “synaptic vesicle membrane” (body: cell component), and “calmodulin binding” (body: molecular function) (Supplementary Figure 2A). In addition, KEGG pathway analysis revealed that the most significant enrichment pathway was “insulin secretion” (Supplementary Figure 2B).

### 3.2. Construction of the Coexpression Network

**Figure 2:**
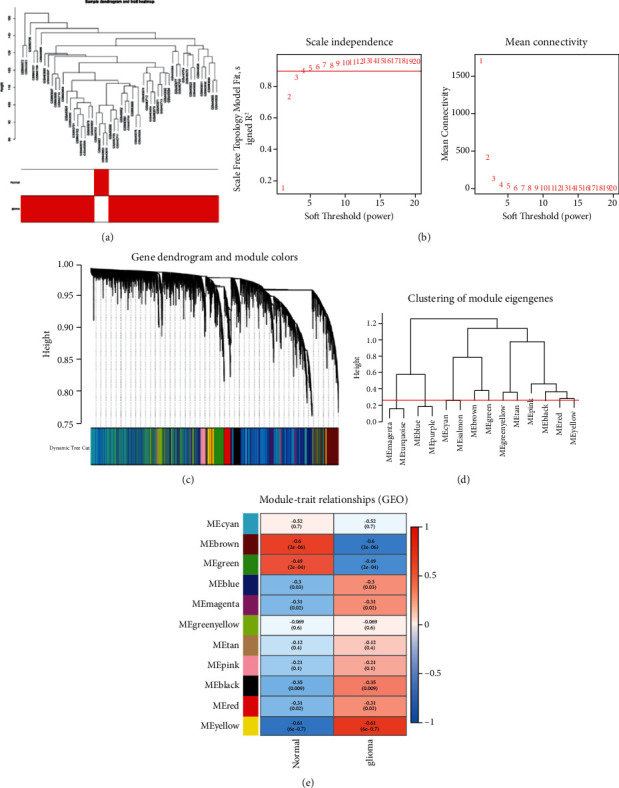
Sample dendrogram and soft-thresholding value estimation. (a) Sample dendrogram and trait heat map. (b) Scale independence and mean connectivity of various soft-thresholding values (*β*). (c) The cluster dendrogram of all filtered genes in the top 25% of variance clustered according to a dissimilarity measure (1-TOM) by WGCNA, which presents 11 gene coexpression modules in GSE2223, which contained 50 glioma and four normal samples. Each branch represents one gene, and every color represents one coexpressed module. WGCNA: weighted gene coexpression network analysis. TOM, topological overlap matrix. (d) Clustering of module eigengenes in GSE2223 by WGCNA. (e) Heat map of the correlations between the clinical traits and MEs of glioma. The rectangles in each row and column represent a module eigengene. In the correlated heat map plot, light blue represents low adjacency, while red represents high adjacency. *p* values are shown. The yellow and brown module showed significant correlation with glioma.

Raw data were normalized using the RMA method in the limma package. Genes with a false discovery rate <0.05 and log2 fold change ≥0.5 were included in the WGCNA analysis. First, genes and samples were examined with the missing values, and all of them met the threshold. Next, the samples were clustered to identify any significant outliers. The height cut-off value was set at 30, and all samples were included in the analysis (Figure 2(a)).

To construct the WGCNA network, the soft threshold power *β* was calculated, and the coexpression similarity was proposed to calculate the adjacency. The pick soft-threshold function in WGCNA was used to analyze the network topology. In subsequent analyses, the soft threshold power *β* was set at 11 due to the scale independence of 0.9 and relatively high average connectivity (Figure 2(b)). The gene network was constructed and modules were identified using the one-step network construction function of the WGCNA R package. For cluster splitting, the soft threshold power was set at 11, minimum module size at 30, and deepSplit at 2 (which correlated with medium sensitivity). Finally, 11 gene coexpression modules were constructed (Figure 2(c)).

### 3.3. Construction of Coexpression Modules and Identification of Key Modules

Relationships between the identified modules were mapped, and the connectivity of eigengenes was analyzed. Eigengenes provide pairings between gene coexpression modules. The results showed that the 11 modules could be clustered into two clusters (Figure 2(d)). The associations between modules and clinical characteristics were evaluated to identify the most significant association. The results of this analysis showed that modules (yellow and brown modules) were most significantly correlated with glioma (correlation coefficients: 0.61 and −0.6, respectively). Therefore, the two modules were selected for further analyses (Figure 2(e)). A scatterplot of gene significance and module membership was plotted in the yellow and brown modules (Supplementary Figure 3).

### 3.4. Identification of Hub Genes

**Figure 3:**
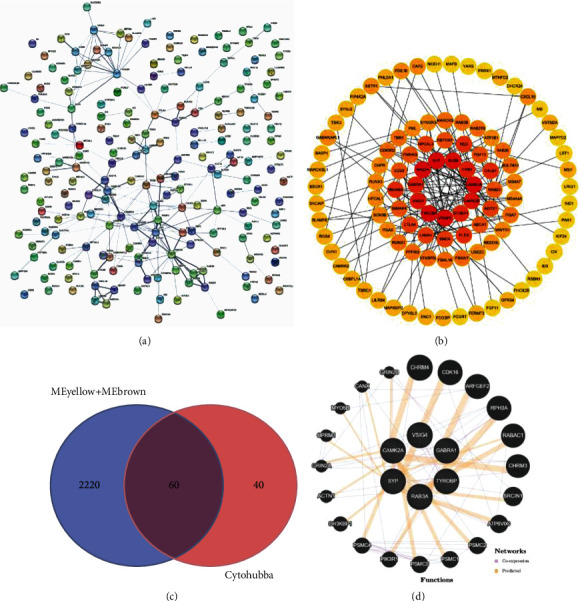
(a) Protein-protein interaction network of DEGs in GSE was constructed using STRING software. (b) The top 100 genes network. The top 100 genes of the degree method were chosen using cytoHubba plugin. The more forward ranking is represented by red color. (c) Overlapping genes of meyellow + mebrown and top 100 hub genes. DEG: differentially expressed genes. (d) Six hub genes and their coexpression genes were analyzed using GeneMANIA. Nodes with white lines represent hub genes. Nodes without white lines represent coexpression genes. Hub genes are determined by the degree of connectivity between differentially expressed genes. The top six hub genes with the highest degree of connectivity were identified, including RAB3A, TYROBP, SYP, CAMK2A, VSIG4, and GABRA1.

To investigate the functions of DEGs, a PPI network was constructed using the STRING database to provide a visual annotation network for identifying the structural and functional properties of proteins (Figure 3(a)). Then, cytoHubba was used to detect the key genes of the PPI network. The top 100 gene networks are shown in Figure 3(b). Using the Venn package in R, the overlapping genes were screened in the yellow-brown module and cytoHubba (Figure 3(c)). We screened 60 overlapping genes; the differences in hub gene expression in the top nine hub genes in glioma are shown in Table 2.

### 3.5. Validation of Hub Gene Expression Levels

**Figure 4:**
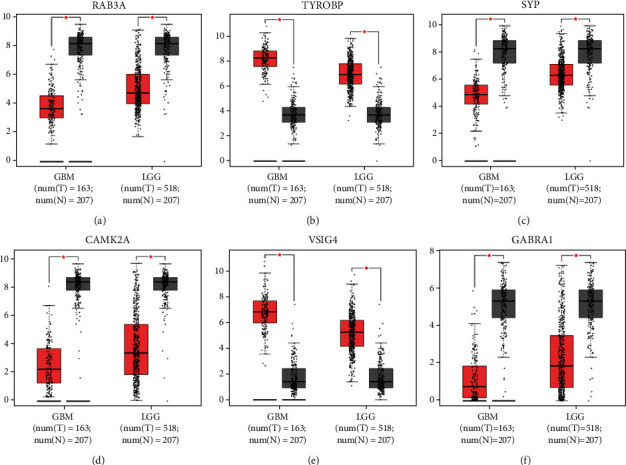
The expression blots for RAB3A, TYROBP, SYP, CAMK2A, VSIG4, and GABRA1 (a–f) from the GEPIA database between glioma and normal brain tissue. Box plots in GEPIA showing that the expression of the six hub genes (RAB3A, TYROBP, SYP, CAMK2A, VSIG4, and GABRA1) was similar to the GSE2223 database (*p* < 0.05). The red node represents tumor samples, and gray node represents normal samples.

The expression levels of the top nine genes in glioma were validated using GEPIA. DEGs between LGG and GBM were considered hub genes. We identified six hub genes: RAB3A, SYP, CAMK2A, and GABRA1 had a lower expression level, while TYROBP and VSIG4 had a higher expression level in 163 GBM and 518 LGG tissues, compared to 207 normal samples (Figure 4). Meanwhile, the results of immunohistochemical staining showed that the expression level of corresponding proteins in glioma tissues was consistent with the transcription level of hub genes selected from the GEO database (Supplementary Figure 4). To explore the relationship between hub genes in glioma, the interaction network of hub genes and their coexpression genes were analyzed using the GeneMANIA online platform (Figure 3(d)).

### 3.6. Expression Level of Hub Genes in the GSE2223 Dataset

**Figure 5:**
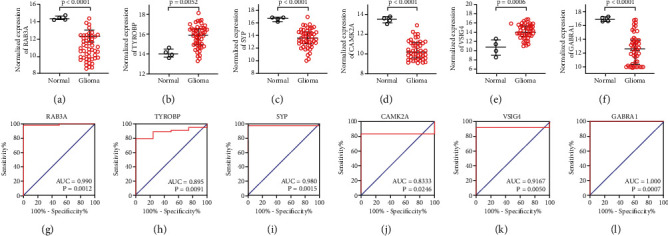
The expression of RAB3A, TYROBP, SYP, CAMK2A, VSIG4, and GABRA1 (a–f) in GSE2223 microarray data. The expression of RAB3A, SYP, CAMK2A, and GABRA1 were decreased, and expression of TYROBP and VSIG4 were increased in glioma samples. ROC curves of RAB3A, TYROBP, SYP, CAMK2A, VSIG4, and GABRA1 (g–l) in GSE2223 microarray data. ROC: receiver operating characteristic.

In the GSE2223 dataset, the expression levels of six hub genes (RAB3A, TYROBP, SYP, CAMK2A, VSIG4, and GABRA1) were consistent with those from the GEPIA database (Figures 5(a)–5(f)). The areas under the curve (AUC) for all six hub genes were ≥0.89, while the receiver operating characteristic (ROC) curve was *p* ≤ 0.03 (Figures 5(g)–5(l), Table 3). Therefore, the six hub genes had a good diagnostic value for glioma.

### 3.7. Prognostic Value of Hub Genes in Glioma

**Figure 6:**
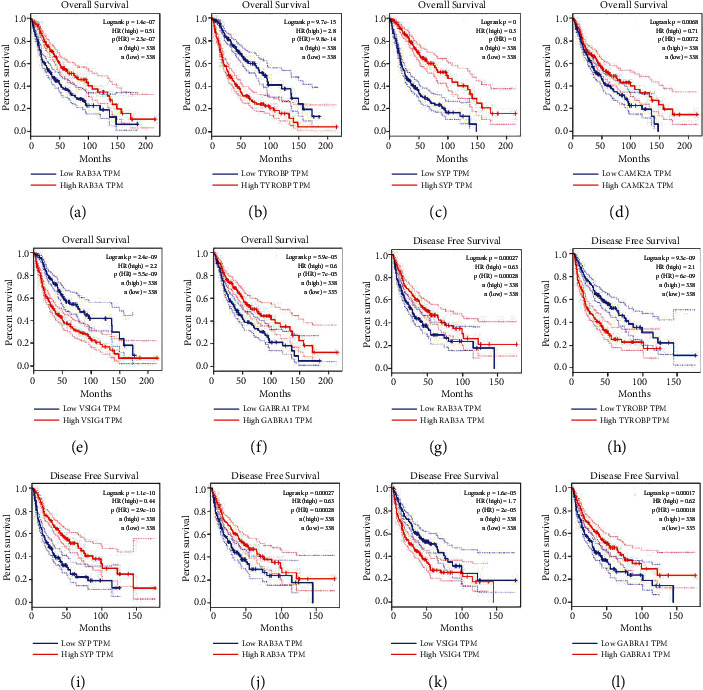
OS for RAB3A, TYROBP, SYP, CAMK2A, VSIG4, and GABRA1 (a–f) from the GEPIA database in glioma patients grouped by median cut-offs. Glioma patients with high expression levels of RAB3A, SYP, CAMK2A, and GABRA1 had improved OS. Glioma patients with high expression levels of TYROBP and VSIG4 were associated with poor OS. OS: overall survival. DFS of RAB3A, TYROBP, SYP, CAMK2A, VSIG4, and GABRA1 (g–l) from the GEPIA database in glioma patients, grouped by median cut-offs. Glioma patients with high expression levels of RAB3A, SYP, CAMK2A, and GABRA1 had improved DFS. Glioma patients with high expression levels of TYROBP and VSIG4 had poor DFS. DFS: disease-free survival.

Using the GEPIA database, the survival curves were produced to explore the prognostic value of hub genes. The higher level of expression of RAB3A, SYP, CAMK2A, and GABRA1 in glioma patients was associated with improved OS and DFS (Figures 6(a), 6(a)-6(d), 6(f)-6(g), 6(i)-6(j), and 6(l)), whereas the higher level of expression of TYROBP and VSIG4 was associated with reduced OS and DFS (Figures 6(b), 6(e), 6(h), and 6(k)). Therefore, the six hub genes had a significant prognostic value in glioma patients and predicted the OS rate and progression-free interval (PFI).

### 3.8. Verification of Hub Gene Expression Levels in Glioma Tissues

**Figure 7:**
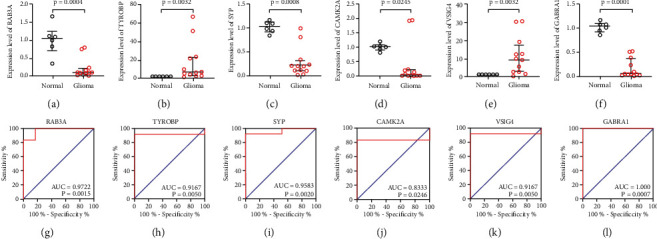
Validation of RAB3A, TYROBP, SYP, CAMK2A, VSIG4, and GABRA1 (a–f) gene expression using real‐time quantitative polymerase chain reaction in glioma samples (normal: 6; glioma: 12). ROC curves for RAB3A, TYROBP, SYP, CAMK2A, VSIG4, and GABRA1 (g–l) in our glioma samples (normal: 6; glioma: 12). ROC: receiver operating characteristic.

The levels of mRNA expression of the hub genes were measured using quantitative PCR in glioma and normal tissues. Compared to the control tissues, glioma tissues had a decreased expression of RAB3A, SYP, CAMK2A, and GABRA1 but an increased expression of TYROBP and VSIG4 (Figures 7(a)–7(f)). These results are in agreement with the findings from the GEO microarray, GEPIA database, and immunohistochemical staining in the Human Protein Atlas database. The AUC and ROC values for the six hub genes were ≥0.83 and *p* ≤ 0.03, respectively. Therefore, all hub genes had a good diagnostic value for glioma. The data from the hub genes were consistent with those from the GSE2223 dataset (Figures 7(g)–7(l), Table 4).

### 3.9. Immune Infiltration Analyses

**Figure 8:**
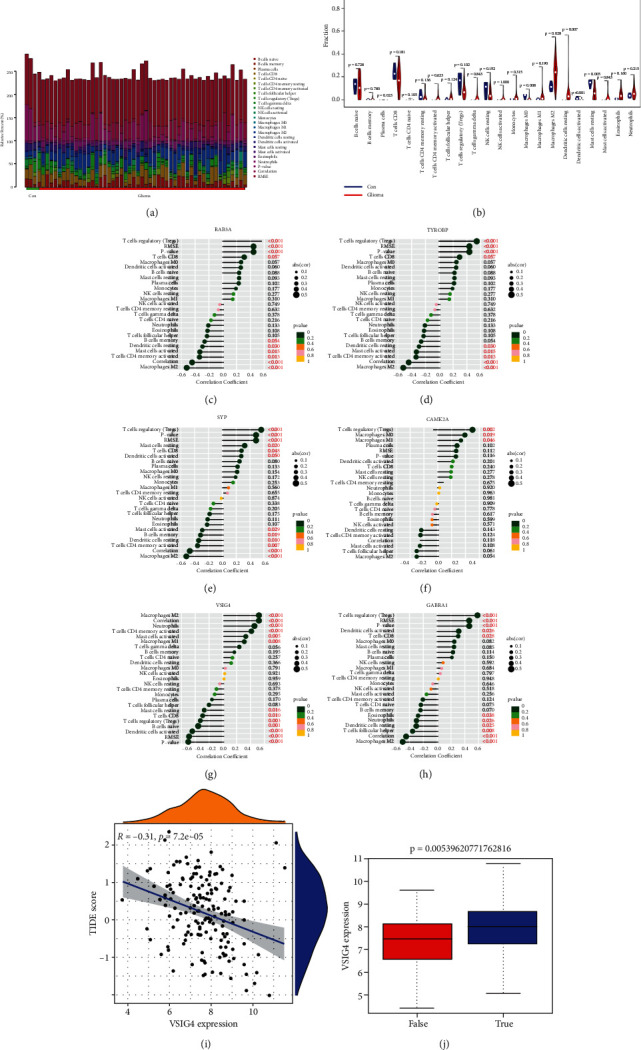
Landscape of immune infiltration between the glioma and control groups. (a) The box plot diagram indicates the relative percentage of different types of immune cells in each sample. (c) The violin plot shows the differences of immune infiltration between the glioma (red) and healthy control (blue) groups. (d) The lollipop chart presents the correlation of RAB3A, TYROBP, SYP, CAMK2A, VSIG4, GABRA1, and infiltrating immune cells on the basis of Spearman correlation analysis results. *p* value <0.05 indicated statistical significance. (d) Correlation between the TIDE score and VSIG4 expression. (e) Differences in expression of VSIG4 to immunotherapy response.

The CIBERSORT algorithm was used to analyze the difference of immune cell infiltration between the glioma and control groups in 22 subpopulations of immune cells. The total value of all immune cells in each sample was set at 100%, and the proportion of each immune cell in these samples is presented in Figure 8(a). The violin plot showed marked differences in the distribution of 13 out of 22 immune cells (Figure 8(b)). Taken together, these results suggest that the heterogeneity of infiltrating immune cells in glioma is evident and may play a role in the pathogenesis of glioma.

To further investigate the correlation of RAB3A, TYROBP, SYP, CAMK2A, VSIG4, GABRA1, and infiltrating immune cells, Spearman correlation was performed and plotted in a lollipop chart (Figures 8(c)–8(h)). These results indicate that the core gene RAB3A, TYROBP, SYP, CAMK2A, VSIG4, and GABRA1 is closely related to the level of immune cell infiltration and plays a crucial role in the immune microenvironment of glioma.

### 3.10. Potential of VSIG4 as an Indicator of Response to Immunotherapy

The TIDE score reflects the sensitivity to immune checkpoint. We evaluated the correlation between the RAB3A, TYROBP, SYP, CAMK2A, VSIG4, and GABRA1 signature and the TIDE score. We only found that the TIDE score was significantly negatively correlated with VSIG4 gene expression (Figure 8(i)). We also observed similar outcomes in VSIG4 high and low subgroups. The high expression levels of the VSIG4 group benefited more from immunotherapy (Figure 8(j)). These findings suggested that patients of the high-expression VSIG4 group may be more sensitive to immunological treatment.

## 4. Discussion

In the present study, we analyzed 50 glioma samples and four normal samples from GSE2223 and constructed a gene coexpression network based on WGCNA. Overlapping genes were confirmed in the yellow-brown module and PPI network of DEGs using the cytoHubba plugin. We identified six hub genes (RAB3A, TYROBP, SYP, CAMK2A, VSIG4, and GABRA1) that predicted the prognosis of glioma. These findings were supported by the RT-qPCR test, which is used in the clinical setting. Finally, the identification of VSIG4 for immunotherapy response in patients with glioma demonstrates utility for immunotherapy research.

After screening a series of databases (GEO, TCGA, HPA, STRING, GEPIA, and GeneMANIA) in GSE2223, 11 modules were identified for glioma progression, followed by six hub genes for prognosis of glioma. RAB3A regulates calcium-dependent lysosome exocytosis and plasma membrane repair through interaction with two effectors: SYTL4 and myosin-9/MYH9 [17]. RAB3A positively regulates acrosome content secretion in sperm cells by interacting with RIMS1 [18, 19]. TYROBP is tyrosine-phosphorylated in the ITAM domain following ligand binding by the associated receptors, which leads to the activation of additional tyrosine kinases and subsequent cell activation [20]. TYROBP stabilizes the TREM2 C-terminal fragment produced by TREM2 ectodomain shedding, which suppresses the release of proinflammatory cytokines [21]. SYP is possibly involved in organizing membrane components and targeting vesicles to the plasma membrane, as well as short-term and long-term synaptic plasticity [22]. CAMK2A regulates dendritic spine development [23] and migration of developing neurons [24]. VSIG4 phagocytic receptor negatively regulates T-cell proliferation and IL-2 production [25]. The RAB18-VSIG4 interaction was involved in reducing glioma proliferation and increasing apoptosis, as well as reducing TMZ sensitivity [26]. Let-7g-5p could inhibit epithelial-mesenchymal transition of glioblastoma by targeting VSIG4, which was consistent with the reduction of the glioma stem cell (GSC) phenotype [27]. High expression of VSIG4 was associated with poor prognosis of OS and PFS in high-grade glioma patients [28]. The GABRA1 ligand-gated chloride channel, which is a component of the heteropentameric receptor for GABA, is the major inhibitory neurotransmitter in the brain [29–31]. GABRA1 plays an important role in the formation of functional inhibitory GABAergic synapses and synaptic inhibition of GABA-gated ion channels [29, 30]. This trend was confirmed by immunohistochemical staining of hub genes in Human Protein Atlas.

The survival curves were produced using the GEPIA database to explore the prognostic value of hub genes. Higher expression levels of RAB3A, SYP, CAMK2A, and GABRA1, as well as lower expression levels of TYROBP and VSIG4, in glioma patients were associated with improved OS and DFS. This suggests that all hub genes have a significant prognostic value in glioma and can predict the OS and PFI event.

The mRNA expression levels for the hub genes in tissue samples were similar to those obtained from the GEO microarray, GEPIA database, and Human Protein Atlas database. AUC values for the hub genes demonstrated a good diagnostic value for glioma, which suggests that they may be useful as diagnostic biomarkers and therapeutic targets in glioma.

In addition, immune infiltration analysis in this study demonstrated that the changes of infiltrating immune cells in glioma are evident. Interestingly, RAB3A, TYROBP, SYP, CAMK2A, VSIG4, and GABRA1 were also found to be closely related to the level of immune cell infiltration in the current study. Therefore, it could be concluded that RAB3A, TYROBP, SYP, CAMK2A, VSIG4, and GABRA1 may play a critical role in glioma by regulating immune cells. We also observed the TIDE score was significantly negatively correlated with VSIG4 gene expression, indicating that the VSIG4 high expression group may be more sensitive to immunological treatment. Different from the classical complement receptors CR3 and CR4, the unique function of VSIG4 suggests unique functions in the regulation of innate and acquired immunity [32]. Soluble Vsig4-IG could attenuate the induction of T cell responses in vivo and inhibit Th cell-dependent responses [25]. VSIG4 signaling inhibited the proliferation of CD4(+) and CD8(+) T cells and the production of IL-2 and IFN-*γ* in coculture in vitro [33]. Besides, after coculture with DCs transfected with hVSIG4 recombinant adenovirus, T cell proliferation potential, cytokine production, and activation marker expression were suppressed [34]. All the above suggests that VSIG4 may contribute to the development of new immunotherapy strategies.

There were some limitations to our study. First, this study included a small sample size. Second, we did not have data for the survival curve analysis (OS and DFS) of glioma patients. Third, the corresponding protein levels for the hub genes were not measured. Fourth, the molecular mechanisms underlying the relationships between hub genes and glioma diagnosis and prognosis were not studied. Therefore, further studies are needed to validate the new therapeutic targets.

## 5. Conclusions

After WGCNA and PPI network analysis of DEGs in glioma samples, a series of multiple databases were screened. Six hub genes related to glioma diagnosis and prognosis were identified, including RAB3A, TYROBP, SYP, CAMK2A, VSIG4, and GABRA1. These genes may help to identify potential therapeutic targets and diagnostic and prognosis biomarkers. We identified VSIG4 for immunotherapy response in patients with glioma, demonstrating utility for immunotherapy research.

## Figures and Tables

**Table 1:** Primer sequences of hub genes.

Gene		Primer (5′ -> 3′)
GAPDH	Forward	GGAGCGAGATCCCTCCAAAAT
	Reverse	GGCTGTTGTCATACTTCTCATGG

RAB3A	Forward	GAGTCCTCGGATCAGAACTTCG
	Reverse	TGTCGTTGCGATAGATGGTCT

TYROBP	Forward	ACTGAGACCGAGTCGCCTTAT
	Reverse	ATACGGCCTCTGTGTGTTGAG

SYP	Forward	CTCGGCTTTGTGAAGGTGCT
	Reverse	CTGAGGTCACTCTCGGTCTTG

CAMK2A	Forward	GCTCTTCGAGGAATTGGGCAA
	Reverse	CCTCTGAGATGCTGTCATGTAGT

VSIG4	Forward	GGGGCACCTAACAGTGGAC
	Reverse	GTCTGAGCCACGTTGTACCAG

GABRA1	Forward	AGCCGTCATTACAAGATGAACTT
	Reverse	TGGTCTCAGGCGATTGTCATAA

**Table 2:** Top nine overlapping genes in GSE2223.

	Gene	logFC	Average expression	*p* value	Adjusted *p* vaue
1	RAB3A	−2.601184259	11.16312569	0.00032	0.008621
2	TYROBP	2.503201759	15.59798543	8.59E-05	0.003944
3	SYP	−2.391063519	13.73489952	0.000715	0.013885
4	CAMK2A	−2.399456944	10.5253732	2.59E-06	0.000669
5	VAMP2	−1.504794074	12.35222771	1.99E-05	0.001808
6	SV2B	−3.216642593	12.1141689	0.00066	0.013313
7	VSIG4	3.576306204	13.8629201	5.78E-05	0.003276
8	CAMK2B	−3.133447315	11.39848628	2.43E-05	0.001964
9	GABRA1	−3.897073333	12.88497377	0.000231	0.007141

**Table 3:** ROC curve validation of 6 hub genes in GSE2223.

	Hub gene	AUC	95% CI	*p* value
1	RAB3A	0.99	0.9662 to 1.000	0.0012
2	TYROBP	0.895	0.8018 to 0.9882	0.0091
3	SYP	0.98	0.9412 to 1.000	0.0015
4	CAMK2A	1	1.000 to 1.000	0.001
5	VSIG4	0.95	0.8791 to 1.000	0.003
6	GABRA1	0.98	0.9439 to 1.000	0.0015

AUC: area under the curve; ROC: receiver operating characteristic; CI: confidence interval.

**Table 4:** ROC validation of 6 hub genes in our glioma samples (6 normal vs. 12 glioma samples).

	Hub gene	AUC	95% CI	*p* value
1	RAB3A	0.9722	0.9034 to 1.000	0.0015
2	TYROBP	0.9167	0.7603 to 1.000	0.005
3	SYP	0.9583	0.8675 to 1.000	0.002
4	CAMK2A	0.8333	0.6225 to 1.000	0.0246
5	VSIG4	0.9167	0.7603 to 1.000	0.005
6	GABRA1	1	1.000 to 1.000	0.0007

AUC: area under the curve; ROC: receiver operating characteristic; CI: confidence interval.

## Data Availability

Publicly available datasets were analyzed in this study. These data can be found in the following link: https://www.ncbi.nlm.nih.gov/sra/?term=SRP049695.

## Supplementary Materials

Supplementary Materials Supplementary Figure 1: screening of differentially expressed genes (DEGs) in GSE2223. (A) Heat map of DEGs (fold change >1.5; adjusted *p* < 0.05). N represents normal brain tissue; T represents glioma tissue. (B) Volcano map of DEGs; red represents upregulated genes, and blue represents downregulated genes. Supplementary Figure 2: top ten DEGS with enrichment of the gene ontology (GO) terms and Kyoto Encyclopedia of Genes and Genomes (KEGG) pathways. (A) GO analysis; (B) KEGG pathways analysis. GO: Gene Ontology; KEGG: Kyoto Encyclopedia of Genes and Genomes. Supplementary Figure 3: scatter plot analysis of yellow modules (A) and brown modules (B). Supplementary Figure 4: representative immunohistochemistry images of the six hub genes, including RAB3A, TYROBP, SYP, CAMK2A, VSIG4, and GABRA1 (A–F), from the Human Protein Atlas.
