# Supplementary material for: Identification of a Signature for Predicting Prognosis and Immunotherapy Response in Patients with Glioma
Source: J Oncol. 2022 Aug 29;2022:8615949. doi: 10.1155/2022/8615949 (PMC9444386; doi:10.1155/2022/8615949)

Supplementary Figure 1. Screening of differentially expressed genes (DEGs) in GSE2223. (A) Heat map of DEGs (fold change > 1.5; adjusted p < 0.05). N represents normal brain tissue; T represents glioma tissue. (B) Volcano map of DEGs; red represents upregulated genes and blue represents downregulated genes.

Supplementary Figure 2. Top ten DEGS with enrichment of the gene ontology (GO) terms and Kyoto Encyclopedia of Genes and Genomes (KEGG) pathways. (A) GO analysis; (B) KEGG pathways analysis. GO: Gene Ontology; KEGG: Kyoto Encyclopedia of Genes and Genomes.

Supplementary Figure 3. Scatter plot analysis of yellow modules (A) and brown modules (B).

Supplementary Figure 4. Representative immunohistochemistry images of the six hub genes, including RAB3A, TYROBP, SYP, CAMK2A, VSIG4, and GABRA1 (A–F). from the Human Protein Atlas.

**Supplementary Figure 1**


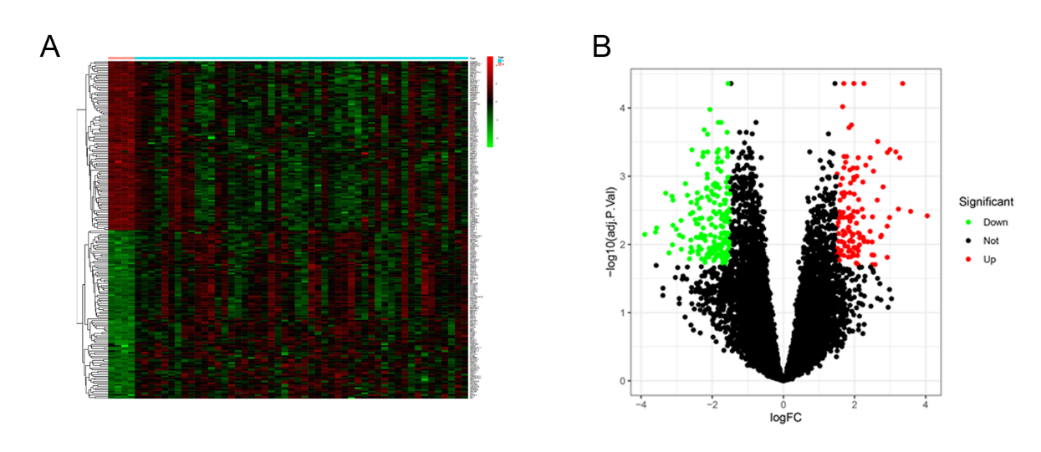


**Supplementary Figure 2**


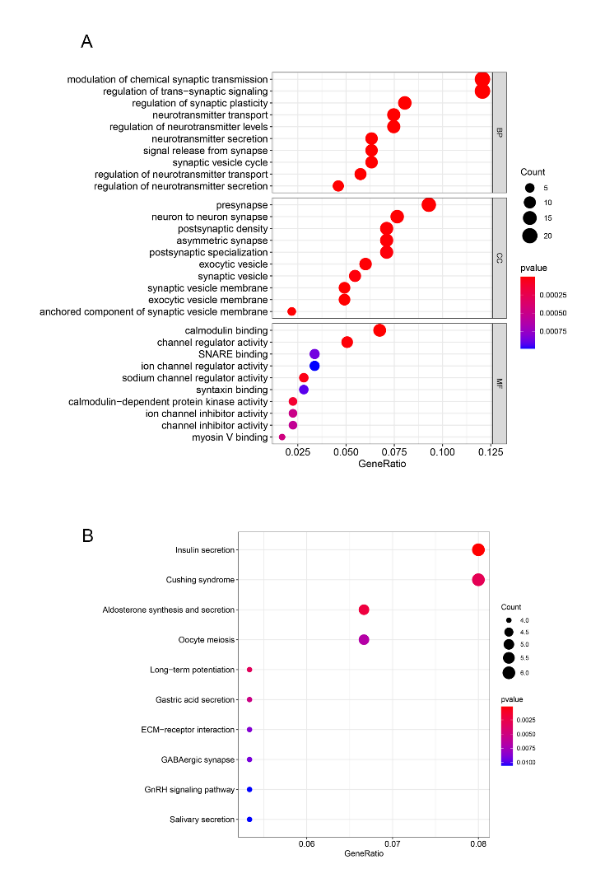


**Supplementary Figure 3**


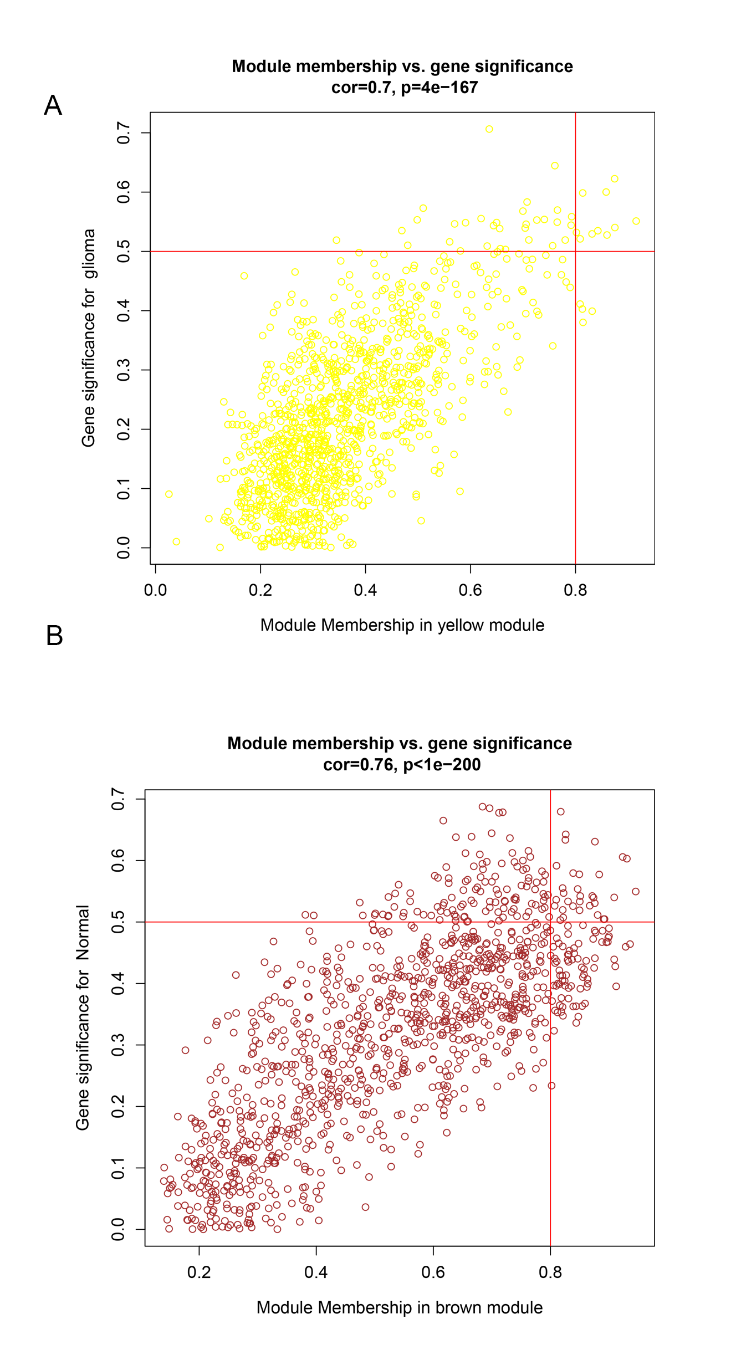


**Supplementary Figure 4**


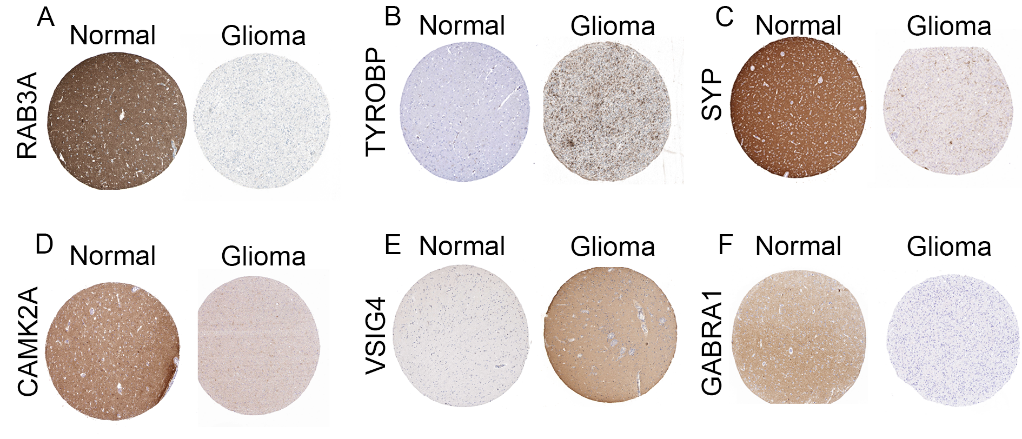

Supplement: Supplementary Materials — Supplementary Figure 1: screening of differentially expressed genes (DEGs) in GSE2223. (A) Heat map of DEGs (fold change >1.5; adjusted p < 0.05). N represents normal brain tissue; T represents glioma tissue. (B) Volcano map of DEGs; red represents upregulated genes, and blue represents downregulated genes. Supplementary Figure 2: top ten DEGS with enrichment of the gene ontology (GO) terms and Kyoto Encyclopedia of Genes and Genomes (KEGG) pathways. (A) GO analysis; (B) KEGG pathways analysis. GO: Gene Ontology; KEGG: Kyoto Encyclopedia of Genes and Genomes. Supplementary Figure 3: scatter plot analysis of yellow modules (A) and brown modules (B). Supplementary Figure 4: representative immunohistochemistry images of the six hub genes, including RAB3A, TYROBP, SYP, CAMK2A, VSIG4, and GABRA1 (A–F), from the Human Protein Atlas. [file 8615949.f1.docx]
